# Loss of Sec-1 Family Domain-Containing 1 (*scfd1*) Causes Severe Cardiac Defects and Endoplasmic Reticulum Stress in Zebrafish

**DOI:** 10.3390/jcdd10100408

**Published:** 2023-09-22

**Authors:** Inken G. Huttner, Celine F. Santiago, Arie Jacoby, Delfine Cheng, Gunjan Trivedi, Stephen Cull, Jasmina Cvetkovska, Renee Chand, Joachim Berger, Peter D. Currie, Kelly A. Smith, Diane Fatkin

**Affiliations:** 1 Molecular Cardiology and Biophysics Division, Victor Chang Cardiac Research Institute, Darlinghurst, NSW 2010, Australia; inkengmartin@gmail.com (I.G.H.); c.santiago@victorchang.edu.au (C.F.S.); arijac68@gmail.com (A.J.); delfine.cheng@victorchang.edu.au (D.C.); g.trivedi@victorchang.edu.au (G.T.); stephengcull@gmail.com (S.C.); j.cvetkovska@victorchang.edu.au (J.C.); r.chand@victorchang.edu.au (R.C.); 2 School of Clinical Medicine, Faculty of Medicine and Health, UNSW Sydney, Kensington, NSW 2052, Australia; 3 Australian Regenerative Medicine Institute, Monash University, Clayton, VIC 3800, Australia; joachim.berger@monash.edu (J.B.); peter.currie@monash.edu (P.D.C.); 4 European Molecular Biology Labs (EMBL) Australia, Victorian Node, Monash University, Clayton, VIC 3800, Australia; 5 Department of Anatomy & Physiology, The University of Melbourne, Parkville, VIC 3010, Australia; kelly.smith1@unimelb.edu.au; 6 Cardiology Department, St Vincent’s Hospital, Darlinghurst, NSW 2010, Australia

**Keywords:** dilated cardiomyopathy, genetics, *scfd1*, zebrafish, ER stress, protein homeostasis

## Abstract

Dilated cardiomyopathy (DCM) is a common heart muscle disorder that frequently leads to heart failure, arrhythmias, and death. While DCM is often heritable, disease-causing mutations are identified in only ~30% of cases. In a forward genetic mutagenesis screen, we identified a novel zebrafish mutant, *heart and head* (*hah*^vcc43^), characterized by early-onset cardiomyopathy and craniofacial defects. Linkage analysis and next-generation sequencing identified a nonsense variant in the highly conserved *scfd1* gene, also known as *sly1*, that encodes sec1 family domain-containing 1. Sec1/Munc18 proteins, such as Scfd1, are involved in membrane fusion regulating endoplasmic reticulum (ER)/Golgi transport. CRISPR/Cas9-engineered *scfd1*^vcc44^ null mutants showed severe cardiac and craniofacial defects and embryonic lethality that recapitulated the phenotype of *hah*^vcc43^ mutants. Electron micrographs of *scfd1*-depleted cardiomyocytes showed reduced myofibril width and sarcomere density, as well as reticular network disorganization and fragmentation of Golgi stacks. Furthermore, quantitative PCR analysis showed upregulation of ER stress response and apoptosis markers. Both heterozygous *hah*^vcc43^ mutants and *scfd1*^vcc44^ mutants survived to adulthood, showing chamber dilation and reduced ventricular contraction. Collectively, our data implicate *scfd1* loss-of-function as the genetic defect at the *hah*^vcc43^ locus and provide new insights into the role of *scfd1* in cardiac development and function.

## 1. Introduction

Dilated cardiomyopathy (DCM) is a common heart muscle disorder, characterized by contractile dysfunction with or without ventricular dilation, and is a leading cause of morbidity and mortality. Over the past two decades, significant progress has been made in deciphering the genetic causes of DCM [1]. Despite these advances however, the yield of genetic testing in patients with suspected familial disease remains low (~30%). Thus, the discovery of novel DCM-causing genes and elucidation of underlying molecular mechanisms is a clinical imperative and rate-limiting step for personalized diagnosis and treatment.

DCM-associated genes are involved in diverse biological processes in cardiomyocytes, including cytoskeletal and sarcomeric structure, force generation and transmission, desmosome structure and function, sensing and transduction of mechanical stress, and mitochondrial function [2,3]. There is also a growing body of evidence that dysregulation of protein homeostasis in the heart, including functional defects in the endoplasmic reticulum (ER) and Golgi apparatus, and activation of ER stress responses may be critically involved in the pathogenesis of genetic cardiomyopathies [4,5,6,7,8].

Zebrafish are a particularly useful animal model for the discovery of new cardiomyopathy genes, due to their embryonic translucency and high genetic and functional conservation to humans [9]. Of more than 3000 potential human disease genes listed in the Online Mendelian Inheritance in Man database, over 2500 (82%) are also present in zebrafish [10]. Forward genetic N-ethyl-N-nitrosourea (ENU) mutagenesis screens have led to the identification of numerous zebrafish genes whose human orthologues were subsequently shown to cause cardiomyopathy, such as *ttn* (*pickwick*) [11], *tnnt2* (*silent heart*) [12], and *mlc2a* (*tell-tale heart*) [13]. Furthermore, advances in high-frequency echocardiographic imaging techniques have opened new avenues for using adult zebrafish to model adult-onset cardiomyopathies, such as titin-related DCM [14].

Here, we performed a phenotype-driven ENU mutagenesis screen and isolated a novel mutant, *heart and head* (*hah*^vcc43^), that manifests cardiac and craniofacial defects. Genome sequencing and linkage analysis mapped the *hah*^vcc43^ mutation to the sec1 family domain-containing 1 (*scfd1*) gene (also known as *sly1*). *scfd1* encodes a member of the Sec1/Munc18 (SM) family of proteins, which cooperate with soluble N-ethylmaleimide-sensitive factor attachment protein receptor (SNARE) complexes in membrane fusion events in the secretory and endo-lysosomal pathways, as well as ER to Golgi ante- and retrograde transport [15,16,17,18]. In zebrafish, Scfd1 was previously shown to be widely expressed, including in the heart, and to play an important role in cartilage formation and fin regeneration [19,20]. While it was observed that *scfd1* loss was associated with embryonic pericardial edema and altered gross cardiac morphology [19,20], detailed functional and ultrastructural evaluation of the heart was not undertaken. We show here that *scfd1* deficiency in embryos leads to a thin-walled ventricular chamber with severely reduced contractility, reduced cardiomyocyte sarcomere content, altered cardiomyocyte ER and Golgi morphology, and upregulation of ER stress and apoptosis markers. In addition, partial *scfd1* deficiency in adulthood results in ventricular systolic dysfunction, suggesting that primary defects in SM proteins may represent a potential novel mechanism for DCM.

## 2. Materials and Methods

### 2.1. Zebrafish Husbandry

Zebrafish (Danio rerio) were raised and maintained according to standard procedures [21]. All experiments using zebrafish were performed in compliance with relevant laws and institutional guidelines and were approved by the Garvan Institute of Medical Research/St Vincent’s Hospital Animal Ethics Committee and the Institutional Biosafety Committee.

### 2.2. Generation of hah^vcc43^ Mutant

As described previously [10], adult male zebrafish were sedated and treated with the chemical mutagen, ENU (Sigma-Aldrich, St. Louis, MO, USA). Mutagenized F0 males were out-crossed to *TE* wildtypes (WT) and resulting F1 offspring were crossed to homozygous *Tg(α-actin:GFP)* fish to establish F2 families with GFP expressed within the skeletal muscle. F2 families were in-crossed and their offspring were screened at 3 days post fertilization (dpf) for the presence of gross cardiac defects and skeletal muscle defects. The identified *hah* mutant was out-crossed to *TE* over 6 generations to reduce background mutations before linkage analysis was performed.

### 2.3. Mapping of hah^vcc43^ Mutation

Heterozygous *hah* mutants were out-crossed to *WIK* WT to establish a mapping cross. To identify the genetic cause of the *hah* phenotype, we used a genome sequencing mapping approach, as previously published [22,23]. Briefly, 45 homozygous *hah*^vcc43^ mutant embryos and 45 siblings at 4 dpf were pooled and homogenized, and genomic DNA was extracted using the Qiagen DNeasy^®^ Blood & Tissue Kit (Qiagen, Hilden, Germany). Library preparation and sequencing were performed by the Institute for Molecular Bioscience Sequencing Facility, University of Queensland, St Lucia, QLD, Australia. Mutant and sibling DNA was converted into libraries using the KAPA HTP Library Preparation Kit (Kapa Biosystems, Wilmington, MA, USA) according to manufacturer’s instructions. Sequencing was performed on the Illumina™ HiSeq 2000 platform (Illumina, Inc., San Diego, CA, USA). Reads were then mapped to the *Danio rerio* reference genome (danRer7/Zv9). Regions of high parent strain homozygosity were identified [24], and candidate variants were detected [22,23].

To prioritize candidate variants, we used web-based in silico prediction tools including two programs that allowed both human and zebrafish sequence input (SIFT ([25] and PMut [26]) and two that permitted human sequence input only (PolyPhen-2 [27] and SNPs&GO [28] [last accessed June 2022]). The impact of zebrafish sequence variants was classified as: high (deleterious in both programs), moderate (deleterious in one and neutral in the other), or low (neutral in both programs). The impact of human sequence variations that corresponded to the zebrafish variants were classified as: high (deleterious in all 4 programs), moderate (deleterious in 2 or 3 of the 4 programs), or low (neutral in 3 of the 4 programs).

Genotyping of *hah*^vcc43^ mutants was performed by polymerase chain reaction (PCR) amplification of genomic DNA extracted from whole embryos or adult fin clips, using the following primers: scfd1_I9-F: 5′-caagttggtggttcattcca; scfd1_I10-R: 5′-ctgttaacgtgccatgccta. This was followed by Sanger sequencing.

### 2.4. Morpholino-Mediated Knockdown of scfd1

Morpholino (MO)-modified antinucleotides (Gene Tools, OR, USA) designed to specifically inhibit mRNA translation of *scfd1* in zebrafish have been published (5′-gtttttcccggatggacgccgccat-3′) [18]. In this experiment, 0.2 mM of either the *scdf1*-MO or a standard control-MO (5′-cctcttacctcagttacaatttata-3′) was injected into fertilized zebrafish eggs at the one-cell stage. Injected embryos were kept at 28 °C and phenotypically evaluated at 3 dpf.

### 2.5. CRISPR—CRISPR-Associated Protein 9 (CAS9)-Mediated Generation of scfd1^vcc44^

Clustered Regularly Interspaced Short Palindromic Repeats (CRISPR)—CRISPR-associated protein 9 (Cas9)-mediated gene editing was used (Alt-R^®^ system by Integrated DNA Technologies, Inc., Coralville, IA, USA) to generate a zebrafish mutant harboring a premature termination codon (PTC) in exon 10 of the *scfd1* gene, which mimics the *hah*^vcc43^ mutation. A custom *scfd1* crisprRNA (crRNA) sequence was identified using the IDT design tool (Integrated DNA Technologies, Inc., Coralville, IA, USA) (5′-tgttcttgcggatcgtaacctgg-3′). In addition to targeting the *scfd1* gene, a second crRNA (5′-cgttgggaaggtcggacaccgttttagagcttgct-3′) was designed (IDT, Coralville, IA, USA) to target the zebrafish tyrosinase gene responsible for pigment formation. Simultaneous targeting of the *tyr* and *scfd1* genes acted as a positive control, with pigment defects in the injected zebrafish indicating likely successful genetic modification of the target gene of interest. Active guideRNAs (gRNAs) were generated by annealing universal tracrRNA (trRNA) (IDT, Coralville, IA, USA) and gene-specific crRNA pairs, and these were then incubated with purified Cas9 nuclease V3 (IDT, Coralville, IA, USA) to obtain active ribonucleoprotein (RNP) complexes. RNPs were microinjected into fertilized one-cell stage WT zebrafish embryos using a Picospritzer III Microinjection System (051-0500-200-003, Parker Hannifin, Hollis, NH, USA). Injected F0 embryos were raised to adulthood and out-crossed. F1 founders were screened for the presence of the desired *scfd1* mutation by Sanger sequencing, followed by analysis using PolyPeakParser (http://yosttools.genetics.utah.edu/PolyPeakParser, accessed on 25 June 2020). The selected F1 founders, harboring an 11-nucleotide deletion that was predicted to lead to a frameshift, 17 codons of neo-sequence followed by a PTC, were out-crossed again, and F2 heterozygous mutant fish were raised to adulthood and in-crossed. F3 embryonic and adult fish were used for phenotypic analyses.

Genotyping of *scfd1*^vcc44^ mutant zebrafish was performed using PCR of genomic DNA extracted from whole embryos or adult fin clips, using the following primers: scfd1_E10-F2: 5′-agacatgttttcctccactctct; scfd1_E10-R2: 5′-gccatccagggaattgagac. This was followed by restriction enzyme digest using PspGI (New England BioLabs, Ipswich, MA, USA) or Sanger sequencing.

### 2.6. Video-Microscopy

Phenotypic evaluation was performed in anesthetized zebrafish embryos at room temperature by video microscopy as described [14], using a Leica DM IL inverted microscope (Leica Microsystems, Wetzlar, Germany) and a Nikon DS-Qi1MC camera (Nikon Instruments, Tokyo, Japan) with NIS Elements AR v.3.1 software (Nikon Instruments). End-diastolic (EDA) and end-systolic (ESA) chamber areas were derived from short (a) and long axis (b) diameters according to the formula: A = π × ½ a × ½ b. Fractional area change (FAC) was derived using the formula: FAC = (EDA-ESA)/EDA [14]. 

### 2.7. Echocardiography

Underwater echocardiography was performed in adult zebrafish aged 6–15 months using the Vevo3100^®^ Imaging Station (VisualSonics, Amsterdam, The Netherlands) equipped with a high-frequency transducer (MS700D) as described [14,29]. Only male fish were used to avoid increased data variability due to sex differences in ventricular dimensions and diastolic function [29]. Two-dimensional (B-Mode) images, color, and pulsed-wave Doppler signals were recorded in long axis view and optimized for either ventricular or atrial assessment, and either atrioventricular inflow or ventricular outflow assessment, respectively. Image analysis was performed using the VevoLab^TM^ analysis software package version 5.7.0 (VisualSonics) [14,29] by a single operator who was blinded to genotype. B-Mode images in the long axis view were used to derive ventricular end-diastolic and end-systolic volumes (EDV, ESV) and maximal atrial size (atrial area (AA)), standardized by indexing to body surface area (BSA). Speckle tracking analysis of ventricular wall motion was performed with the VevoStrainTM analysis software package version 5.7.0 (VisualSonics) and was used to calculate the ejection fraction (EF) and global longitudinal strain (GLS).

### 2.8. qPCR

Total RNA from pooled *TE* WT, as well as hetero- and homozygous *hah*^vcc43^ and *scfd1*^vcc44^ mutant embryos (30 embryos/sample, n = 5 samples) were extracted using TRIzol (Sigma-Aldrich, St. Louis, MO, USA) and the RNeasy Micro Kit (Qiagen, Hilden, Germany) as described [30]. Purified RNA (1000 ng) was used to generate cDNA using the Superscript III First-Strand Synthesis System (Invitrogen, Burlingame, CA, USA). Relative qPCR was carried out in 384-well plates using a Light Cycler 480 thermal cycler (Roche, Basel, Switzerland), with the primers listed in Supplementary Table S1. Gene expression was normalized to the expression level of the housekeeper genes *tmem50a* and *ube2a* using ΔΔCt (cycle threshold) values, and graphed relative to transcript expression in WT fish.

### 2.9. mRNA Injection/Rescue

A WT *scfd1* cDNA clone was purchased from Genscript (Piscataway, NJ, USA; Clone ID:ODa02168C). For injections, invitro transcribed mRNA was prepared using the mMessage mMachine Kit (ThermoFisher, Waltham, MA, USA; Ambion #AM1344). The transcribed *scfd1* mRNA was purified using the precipitation method with NaOAC (pH 5.2) and ethanol. Embryos were generated by in-crossing of adult heterozygous *hah*^vcc43^ mutants and injected at the one-cell stage as previously described [18]. Pressure was adjusted to inject approximately 1 nL of RNA at concentrations of 150, 300, and 600 ng/µL, or dye solution as control. Injected embryos were observed daily and scored at 3 and 4 dpf for the presence of pericardial edema, reduced cardiac contractility, small eyes, and an abnormal lower jaw. Phenotypes were classified as: severe (presence of all four mutant features); mild (presence of small eyes and/or jaw in absence of cardiac abnormalities), or normal (absence of all mutant features).

### 2.10. Western Blot

Embryos were pooled at 3 dpf, de-yolked using a calcium-free buffer (1.7 g NaCl, 0.15 g KCl, 0.2 g NaHCO3, in 500 mL H2O (pH to 7)) (30 embryos/sample, n = 6 samples), and homogenized on ice in 1× RIPA buffer containing protease and phosphatase inhibitors (Sigma Aldrich, St. Louis, MO, USA, #PPC1010). Protein concentration was quantified and samples were loaded onto a NuPAGE 4–12% Bis-Tris mini-protein gel (Invitrogen, Burlingame, CA, USA, NP0330) at a concentration of 15 µg under denatured and reduced conditions. After electrophoresis, proteins were transferred onto 0.2 µm PVDF membranes, which were blocked in 10% skim milk-0.1% TBST, incubated in primary antibody overnight at 4 °C and secondary antibody for 2 h at room temperature (RT) (antibodies diluted in 5% skim milk-0.1% TBST). Antibodies: SCFD1 rabbit polyclonal (1:500, ThermoFisher, #PA5-51966), α-Tubulin mouse monoclonal (loading control, 1:3000, Sigma-Aldrich, #T9026), anti-rabbit horse radish peroxidase (HRP) (1:2500, Bio-Strategy, #NA934), anti-mouse HRP (1:15,000, Cytiva, #GENA931). Enhanced chemiluminescence was used to detect bands of interest (Pierce^TM^ ECL Western Blotting Substrate, ThermoFisher, #32106). Immunoblotted PVDF membranes were imaged using a BioRad ChemiDoc Imager and densitometry was performed using ImageLab software version 6 (BioRad, Hercules, CA, USA). Scfd1 protein levels were normalized to α-Tubulin as a loading control.

### 2.11. Electron Microscopy

Embryos were collected at 3 dpf and fixed in 4% paraformaldehyde + 2.5% glutaraldehyde in 0.1 M cacodylate buffer (containing 4% sucrose, 0.15 mM CaCl_2_) for 1 h at RT (n = 3 per genotype). Following 3 washes with 0.1 M cacodylate buffer, the samples were processed for electron microscopy in a BioWave microwave oven (Pelco, Fresno, CA, USA) following a protocol derived from [31,32] in which samples were impregnated with heavy metal stains, namely 2% osmium tetroxide, 1% potassium ferricyanide, 1% thiocarbohydrazide, 2% osmium tetroxide, 1% uranyl acetate and lead aspartate. Finally, the samples were infiltrated in procure resin over 3 days, embedded in fresh resin, and polymerized at 60 deg over 2 days. Ultra-thin sections of 60 nm were generated using an ultramicrotome (Ultracut 7, Leica Microsystems, Wetzlar, Germany), collected on 200 m mesh grids, and imaged using a transmitted electron microscope at 100 kV (JEM-1400, JEOL).

To quantify sarcomere content (number of Z-discs per 80 µm^2^ regions of interest [ROI]) and myofibril width (width of individual Z-discs across the myofibril bundle), images from WT (n = 24) and *scfd1^vcc44^*^−/−^ mutant (n = 29) ventricular cardiomyocytes from 3 embryos per genotype were used. For each fish, 8–10 ROIs from different sections of the ventricle were analyzed, Z-disc numbers/ROI were averaged, and results were plotted. For myofibril width, the width of a total of 116–129 individual Z-discs from all ROIs was measured for each fish, and results were plotted. For quantification of Golgi apparatus morphology, the number of Golgi cisternae, as well as the number of Golgi vesicles immediately surrounding these cisternae, were counted for each Golgi stack (WT: n = 14, *scfd1^vcc44^*^−/−^ mutant: n = 23) on electron micrographs of ventricular cardiomyocytes from 3 embryos per genotype. For each fish, 4–5 individual Golgi stacks were analyzed in WT and 7–8 Golgi stacks in *scfd1^vcc44^*^−/−^ mutant embryos. Cisternae and vesicle numbers were plotted in scatter graphs individually and expressed as a vesicle/cisternae ratio. For sarcomere and Golgi analyses, the examiner was blinded to genotype.

### 2.12. Statistical Analysis

Differences between the expected and observed frequencies in phenotype distribution in embryos following *scfd1* mRNA injection were determined using chi-square tests. *scfd1* transcript and protein expression in groups of WT and mutant fish was assessed using one-way ANOVA analysis with multiple comparisons. Embryonic fish survival was assessed using the log-rank (Cox-Mantel) test. Embryonic heart function at different time points was assessed using two-way ANOVA with multiple comparisons. Echocardiographic analysis of adult heart function was assessed using Brown-Forsythe & Welch ANOVA tests. Mann-Whitney tests were used to calculate the significance of differences between and WT and *scfd1^vcc44^*^−/−^ embryos for abnormally distributed variables (Golgi and sarcomere phenotypes), and Student’s t-test with Welch correction was used for comparison of ER stress markers. Data were expressed as mean ± SD. Statistical analyses were performed in GraphPad Prism 8 (San Diego, CA, USA). Significance if *p* < 0.05. Figures were created with BioRender.com.

## 3. Results

### 3.1. Identification of scfd1 as a Candidate Gene at the hah^vcc43^ Locus

**Figure 1:**
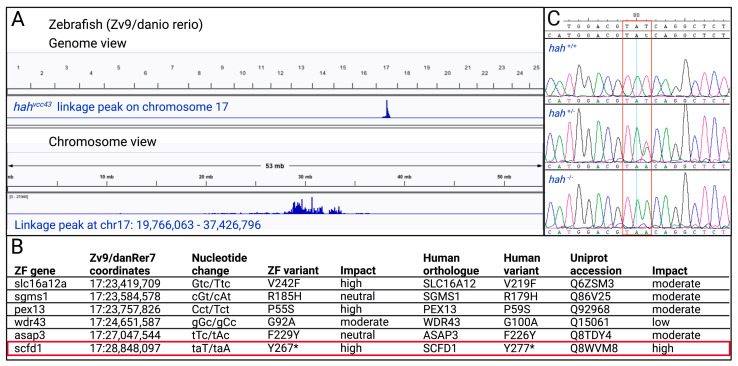
Mapping of zebrafish (ZF) *hah^vcc43^* mutant. (**A**) Mapping of ENU mutagenesis-induced *hah^vcc43^* mutant to a locus on chromosome 17. The top panel shows the genome view and the bottom panel shows the chromosome view of linkage peak. (**B**) Candidate gene variants in linkage interval, with *scfd1* variant highlighted in red box. Human variants based on full length transcripts in Ensembl. Predicted impact of amino acid variants assessed using SIFT, PMut, PolyPhen-2, and SNPs&GO. (**C**) Sanger sequencing confirms taT > taA codon change (red box) in ZF *scfd1* gene in heterozygous (+/−) and homozygous (−/−) *hah^vcc43^* mutants. Created with BioRender.com.

From a previously described forward genetic ENU mutagenesis screen [33], we identified a zebrafish mutant characterized by pericardial edema, a weakly contracting heart, and reduced head and jaw size at 3 dpf. This mutant was named *heart and head* (*hah*^vcc43^). Genome sequencing and linkage analysis [22,23,24] of *hah*^vcc43−/−^ embryos identified a single 18 Mb linkage peak on chromosome 17 (Figure 1A). This interval is syntenic to a region on human chromosome 14q12 and contained several single nucleotide sequence variants (Figure 1B). A nonsense variant (zebrafish NM_182861.1 c.801T > A, p.Y267*) in exon 10 (of 25) of the sec1family domain-containing 1 (*scfd1*) gene, also known as *sly1*, was the most promising candidate after consideration of variant type, conservation, and predicted impact (Figure 1B). Sanger sequencing of WT, sibling, and mutant embryo DNA confirmed the T > A nucleotide change that was homozygous exclusively in the mutant population (Figure 1C). On the protein level, zebrafish Scfd1 shows very high sequence homology to human and mouse SCFD1 (88% and 87% identity, respectively; Supplementary Figure S1) and is abundantly expressed during zebrafish development [19].

**Figure 2:**
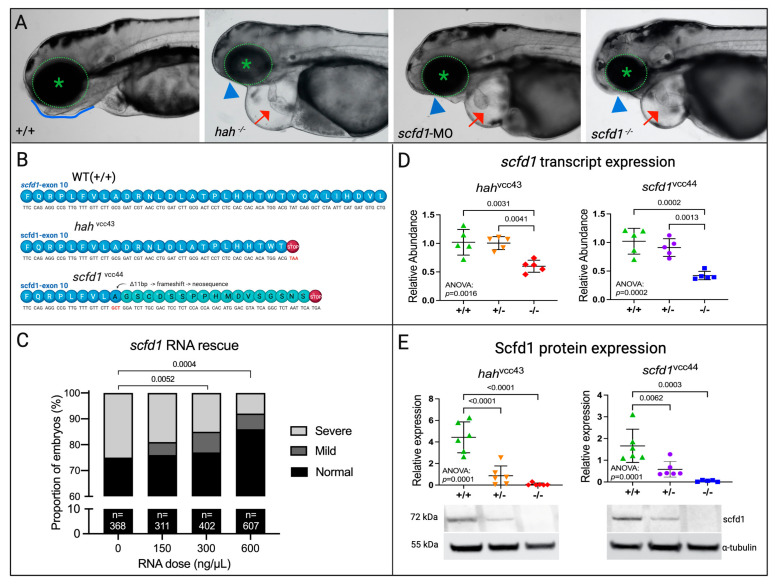
*scfd1* deficiency is responsible for the *hah^vcc43^* mutant phenotype. (**A**) Brightfield photographs show similar phenotypes in *hah^vcc43−/−^* mutants (middle left), *scfd1* morphants (middle right), and *scfd1^vcc44−/−^* mutants (right) in comparison to wildtype (WT; (+/+; left) at 3 days post fertilization (dpf). These include cardiac effusion and poorly ballooned chambers (red arrow), as well as craniofacial defects such as an absence of lower jaw (blue arrowheads) in comparison to a clearly defined jawline in +/+ (blue outline) and small eyes (green asterisk in dotted circle). (**B**) Both *hah^vcc43^* and *scfd1^vcc44^* mutants harbor a PTC in exon 10 of *scfd1*. (**C**) Bar graph showing the proportion of *hah* embryos exhibiting normal (black bars), mild (dark gray bars), or severe (light gray bars) mutant phenotypes at 4 dpf after injection with WT *scfd1* mRNA. Note reduction in proportion of affected embryos with increasing concentrations of *scfd1* mRNA. See Methods for definition of phenotypes. Numbers of embryos in each group indicated at base of bar. Chi-square *p*-values stated. (**D**,**E**) Scatter plots showing reduced *scfd1* transcript expression as determined by qRT-PCR (**D**; 30 embryos/sample, n = 5 samples) and protein expression as determined using Western blot (**E**; 30 embryos/sample, n = 6 samples) in both heterozygous (+/−) and homozygous (−/−) *hah^vcc43^* and *scfd1^vcc44^* mutants at 3 dpf. Ordinary one-way ANOVA *p*-values stated. Significance if *p* < 0.05. α-tubulin loading control used for normalization for Western blot quantification. Full blots are shown in Supplementary Figure S2. Created with BioRender.com.

Analyzing the *hah*^vcc43^ phenotype using light microscopy, *hah*^−/−^ mutants had smaller craniums and eyes, and abnormal jaw development, in comparison with WT embryos at 3 dpf (Figure 2B, top panels, black arrowhead). These craniofacial abnormalities are in accordance with Hou et al. who recovered a zebrafish *scfd1* null mutant from a gene trapping screen [19]. In addition, we found that *hah*^−/−^ embryos displayed prominent cardiac defects, characterized by poorly ballooned and poorly contractile chambers with marked pericardial edema (Figure 2A, middle left panel, red arrow). These features resembled the embryonic phenotype induced by complete loss of the zebrafish genes *ttn*, *tnnt2*, and *mlc2* [11,12,13,14] that are orthologues of human DCM genes. Early cardiac development, including heart tube formation and looping, occurred normally in *hah*^−/−^ mutants. Chamber specification, as indicated by appropriate atrial and ventricular myosin expression, was not significantly altered.

### 3.2. A CRISPR-Cas9-Engineered scfd1 Null Mutant Phenocopies hah^vcc43^

Three approaches were used to provide further genetic evidence for *scfd1*-deficiency as the cause of the *hah*^vcc43^ phenotype: (i) MO antisense oligonucleotide-mediated knockdown of *scfd1* [18], (ii) generation of a stable *scfd1*^vcc44^ mutant line harboring a PTC in exon 10 of *scfd1* (Figure 2B) using CRISPR-Cas9 technology, and (iii) rescue of the *hah*^vcc43^ phenotype using *scfd1* mRNA injection (Figure 2C). Both *scfd1* morphant and *scfd1*^vcc44−/−^ mutant embryos were found to replicate *hah*^vcc43−/−^ craniofacial and cardiac phenotypes (Figure 2B, right panels). Furthermore, injection of increasing concentrations of WT *scfd1* mRNA into 1-cell stage embryos generated by in-crossing of adult heterozygous *hah*^vcc43^ mutants lead to a dose-dependent partial rescue of the *hah*^vcc43^ mutant phenotype (Figure 2C). Further, 25% (92/368) of control-injected embryos had pericardial edema, weak cardiac contractility, small eyes, and small/absent lower jaw at 4 dpf, in line with mendelian inheritance. In contrast, only 8.1% (49/607) of embryos injected with 600 ng/µL *scfd1* mRNA displayed at least 3 of these mutant features, while 91.9% (558/607) were either normal (529/607; 87.1%) or showed small eyes/jaws but normal hearts at 4 dpf (29/607; 4.8%. Chi-square *p* = 0.0004). These findings are in line with the partial *scfd1* mRNA rescue described previously [20]. Together, these observations provide clear evidence that *scfd1* deficiency is responsible for the *hah* phenotype. We next sought to assess if the variants in exon 10 of *scfd1* in our two mutant lines lead to Scfd1 loss due to nonsense-mediated mRNA decay or dominant negative effects through the stable expression of truncated Scfd1 proteins. *scfd1* transcript expression, assessed by qPCR, was relatively unchanged in heterozygous *hah*^vcc43+/−^ and *scfd1*^vcc44+/−^ mutants, but was reduced to approximately 50% in homozygous *hah*^vcc43−/−^ and *scfd1*^vcc44−/−^ mutants (Figure 2D), suggesting nonsense-mediated decay. At the protein level, Western blot analysis using a rabbit polyclonal SCFD1 antibody with zebrafish cross-reactivity (and raised against an N-terminal epitope of the protein unaffected by the predicted truncation; Supplementary Figure S1) showed >50% reduction of Scfd1 protein in heterozygous mutants from both lines, while there was complete loss of Scfd1 in homozygous mutants (Figure 2E). We found no evidence of truncated Scfd1 protein at the expected size, suggesting that *hah*^vcc43−/−^ and *scfd1*^vcc44−/−^ are likely null mutants (Supplementary Figure S2).

### 3.3. Scfd1 Loss Leads to Cardiac Dysfunction in Embryonic Zebrafish

**Figure 3:**
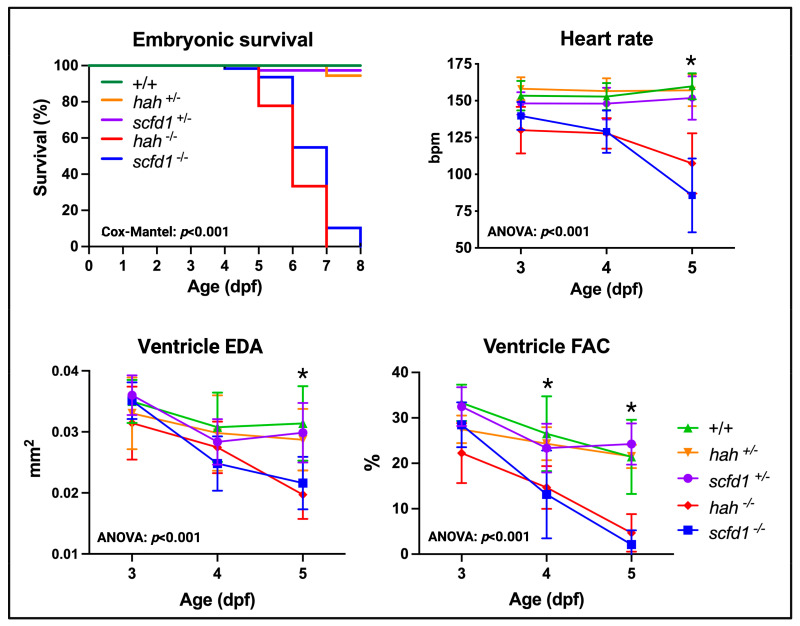
Scfd1 deficiency leads to cardiac dysfunction in zebrafish embryos. Survival, heart rate, ventricular end-diastolic area (EDA) and fractional area change (FAC) are reduced in embryonic *hah^vcc43−/−^* and *scfd1^vcc44−/−^* mutants, while heterozygous siblings of both lines (+/−) are indistinguishable from wildtype (WT; +/+). Non-parametric survival test (Cox-Mantel) and two-way ANOVA *p*-values stated. Animal numbers, mean values, and multiple comparison *p*-values for all variables, time points, and genotypes listed in Supplementary Table S2; asterisk indicates significant differences from WT in *hah^vcc4 −/−^* and *scfd1^vcc44−/−^* mutants. Significance if *p* < 0.05. Created with BioRender.com.

To determine the impact of Scfd1 deficiency in embryonic hearts, video microscopy was used to assess survival, heart rate, ventricular size, and contractility in heterozygous and homozygous *hah*^vcc43^ and *scfd1*^vcc44^ mutant embryos (Figure 3). In comparison to WT, homozygous mutants showed early embryonic lethality by 8 dpf (survival: WT, 96.9%, hah ^vcc43+/−^, 94.4%; hah ^vcc43−/−^, 0%; scfd1 ^vcc44+/−^, 97.4%; scfd1 ^vcc44−/−^, 0%; Mantel-Cox *p* < 0.0001). Furthermore, homozygous mutants from both lines showed progressive bradycardia (two-way ANOVA: genotype factor *p* < 0.0001; multiple comparisons: WT vs. *hah*^vcc43−/−^, *p* = 0.009; WT vs. *scfd1*^vcc44−/−^, *p* = 0.004), as well as reduced ventricular size, as indicated by smaller EDA (two-way ANOVA: genotype factor *p* < 0.0001; multiple comparisons: WT vs. *hah*^vcc43−/−^; WT vs. *scfd1*^vcc44−/−^; *p* < 0.0001), and a significant reduction of ventricular contractility, as indicated by reduced FAC (two-way ANOVA: genotype factor *p* < 0.0001; multiple comparisons: WT vs. *hah*^vcc43−/−^; WT vs. *scfd1*^vcc44−/−^; *p* < 0.0001) at 5 dpf (Figure 3, Supplementary Table S2). Interestingly, heterozygous *hah*^vcc43+/−^ and *scfd1*^vcc44+/−^ mutant embryos were indistinguishable from WT with respect to survival, craniofacial morphology, and cardiac function. In adulthood, heterozygous *hah*^vcc43+/−^ and *scfd1*^vcc44+/−^ mutants displayed normal movement, feeding, and breeding behavior.

### 3.4. Electron Microscopy Showed Reduced Myofibril Width and Sarcomere Density, as well as Abnormal Golgi Apparatus and Reticular Network Morphology in scfd1^vcc44−/−^ Mutant Embryos

To investigate the cause of impaired cardiac function in *scfd1*-deficient fish, we performed electron microscopy on WT and *scfd1*^vcc44−/−^ mutant embryos at 3 dpf. Scfd1 loss in zebrafish has previously been associated with craniofacial abnormalities due to disrupted export of collagen II through the ER-Golgi network, leading to chondrocyte apoptosis and improper jaw cartilage formation [19]. Hence, we first assessed craniofacial chondrocyte ultrastructure in WT and *scfd1*^vcc44−/−^ mutants. In keeping with previous findings, the ER lamellae in WT chondrocytes appeared organized and the extracellular matrix contained a dense network of collagen fibers (Supplementary Figure S3, left panels), while this structure was entirely lost in *scfd1*^vcc44−/−^ mutant chondrocytes (Supplementary Figure S3, right panels). ER tubules appeared disordered, in some parts extended and in others vesiculated, and there was a reduced amount of extracellular matrix, which appeared to contain aggregated rounded fiber remnants.

**Figure 4:**
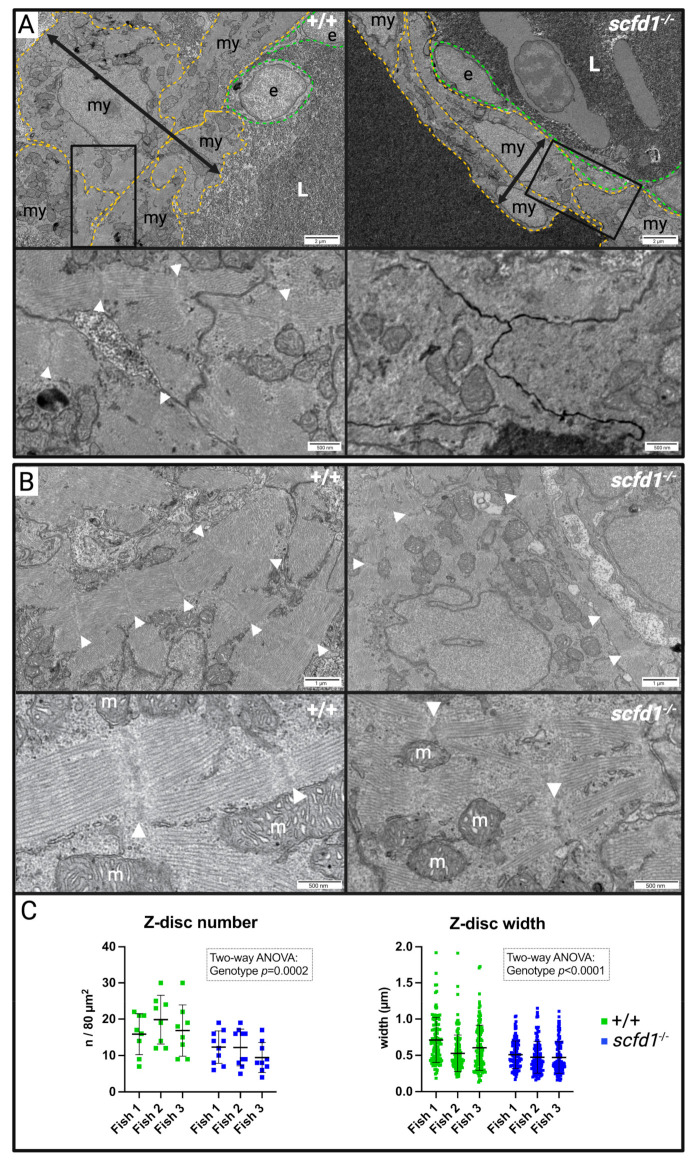
Homozygous *scfd1^vcc44−/−^* mutants show cardiomyocyte ultrastructure defects at 3 dpf. (**A**) Representative low (upper panels) and high (lower panels) magnification electron micrographs of cross-sections of the thinnest part of the ventricular wall illustrating reduced wall thickness in *scfd1* ^vcc44*−/−*^ mutants (black two-headed arrows). my: myocardial cell (yellow outline); e: endocardial cell (green outline); L: lumen. White arrow heads point to sarcomere z-discs. (**B**) Representative low (upper panels) and high (lower panels) magnification electron micrographs of cardiomyocyte myofibrils. White arrow heads point to sarcomere z-discs. m = mitochondrium. (**C**) Scatter graphs quantifying z-disc number (per 80 µm^2^ region of interest (ROI); n = 8–10 ROIs per fish per genotype) and z-disc width (n = 116–129 measurements per fish per genotype) in wildtype (+/+) and *scfd1^−/−^* mutant cardiomyocytes. Results analyzed using two-way ANOVA showing significant genotype effects. Significance if *p* < 0.05. Created with BioRender.com.

Next, we assessed the skeletal and cardiac muscle ultrastructure. Skeletal muscle sarcomere structure appeared normal in both *scfd1*^vcc44−/−^ mutants and WT embryos (Supplementary Figure S4). However, *scfd1*^vcc44−/−^ mutant hearts showed marked abnormalities (Figure 4). Ventricular wall thickness in mutants appeared globally reduced, with cardiomyocytes in the thinnest section of the wall appearing to be elongated and to contain less sarcomeres (Figure 4A). Areas with thick myofibrils consisting of sarcomeres with densely packed, parallel filaments and clearly discernible z-discs were more commonly found in WT cardiomyocytes (Figure 4B, left panels), whereas mutant myofibrils were frequently short and thin, more spread out within the cardiomyocyte, and sarcomere structure appeared less organized (Figure 4B, right panels). Indeed, when quantifying sarcomere content (Z-disc number/ROI) and myofibril width (Z-disc width), there were significant genotype differences (two-way ANOVA: genotype effect *p* = 0.0002 and *p* < 0.0001, respectively; Figure 4C).

**Figure 5:**
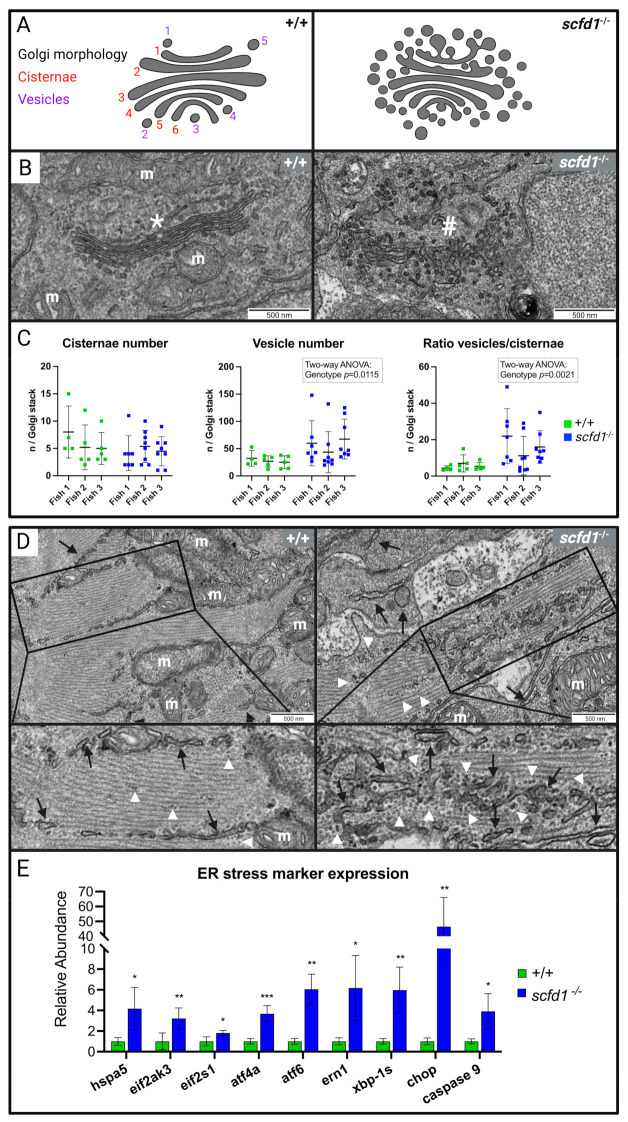
Altered cardiomyocyte Golgi apparatus and reticular network morphology, and upregulation of endoplasmic reticulum (ER) stress markers in *scfd1* mutant hearts at 3 dpf. (**A**) Schematic of normal (left, wildtype (WT; +/+) and altered (right, *scfd1*^vcc44−/−^) Golgi morphology with delineation of Golgi cisternae and vesicles. (**B**) High magnification electron micrographs showing representative examples of ordered Golgi stack (*) in +/+ (left) and highly vesiculated Golgi stack (#) in *scfd1* mutant (right); m = mitochondria. (**C**) Scatter graphs showing increased vesiculation and vesicle/cisternae ratio in Golgi stacks of *scfd1* mutants in comparison to +/+ (n = 4–5 Golgi stacks per fish in +/+, n = 7–8 Golgi stacks per fish in *scfd1*^−/−^). (**D**) Low and high magnification electron micrographs showing normal smooth ER/sarcoplasmic reticulum (SR; black arrows) with occasional clusters of free ribosomes (white arrow heads) in WT cardiomyocytes (left) vs. fractionated, dispersed, and variably sized ER/SR (black arrows), and increased presence of free and membrane-bound ribosomes (white arrow heads) in *scfd1*^vcc44*−/−*^ mutants (right). (**E**) Column graph showing significantly elevated transcriptional expression of ER stress markers in *scfd1*^vcc44−/−^ embryos relative to their expression in WT; n = 5 samples of 30 pooled 3 dpf embryos per genotype. Zebrafish orthologs and mammalian counterparts: hspa5 = GRP78, eif2ak3 = PERK, eif2s-1 = eIF2a, atf4a = ATF4, atf6 = ATF6, ern1 = IRE1. * if *p*= 0.05-0.001; ** if *p*= 0.001-0.0001; *** if *p*<0.0001. Created with BioRender.com.

While structurally connected, the ER and SR form functionally distinct endomembrane compartments in the heart, with the former mainly responsible for protein synthesis, folding, and transport, while the latter acts as a calcium store, regulating cardiomyocyte contraction and electromechanical coupling [34,35,36]. Thus, given the findings in *scfd1*^vcc44−/−^ mutant chondrocytes [19], we next assessed cardiomyocyte endo- and sarcoplasmic reticulum (SR) and Golgi apparatus ultrastructure in detail. In keeping with Scfd1′s proposed role in membrane fusion events required for ER-Golgi vesicle transport, we found that cardiomyocyte Golgi morphology in *scfd1*^vcc44−/−^ mutants was significantly altered in comparison to WT fish (Figure 5A–C). Mutant Golgi apparatuses appeared enlarged and significantly more vesiculated. In some cases, vesicle clusters completely replaced normal Golgi cisternae, resulting in a significantly higher vesicle-to-cisternae ratio in *scfd1*^vcc44−/−^ mutants (Figure 5C; Quantification of vesicle number and vesicle/cisternae ratio: two-way ANOVA: genotype effects *p* = 0.012 and *p* = 0.002, respectively). The ultrastructure of the ER/SR reticular network is variable, and quantification of differences can be challenging. In WT cardiomyocytes, ER/SR tubules were mainly visible at the outer edge of sarcomeres in the inter-myofibrillar region, concentrated near the z-disc and around mitochondria. Rough ER was rarely found outside the perinuclear area, although there were occasional clusters of free ribosomes (Figure 5D, left panels). In contrast, in *scfd1*^vcc44−/−^ cardiomyocytes, there appeared to be areas with a high density of fractionated of ER/SR tubules throughout sarcomeres, interspersed with increased free and membrane-bound ribosomes (Figure 5D, right panels). There were no gross abnormalities in mitochondrial morphology. Together, these findings indicate a significant disruption of the ER-Golgi membrane network in *scfd1*^vcc44−/−^ cardiomyocytes. Interestingly, similar ultrastructural changes to the ER and Golgi networks were also observed in non-cardiomyocyte interstitial cells in the heart.

### 3.5. Increased ER Stress Responses in scfd1^vcc44−/−^ Embryos

To investigate if the observed structural defects in the cardiomyocyte ER-Golgi network led to functional defects, we evaluated the activity of the ER stress response (or unfolded protein response [UPR]) system using qRT-PCR. ER stress results in the upregulation of certain chaperone and signal transduction proteins, as well as activation of ER-associated degradation (ERAD), with the aim of restoring protein homeostasis. ER stress that persists over longer periods can lead to the initiation of apoptosis [7,8,37,38]. Indeed, we found significant transcriptional upregulation of *hspa5*, the zebrafish ortholog of the human glucose-regulating protein 78 (*GRP78*) gene. In addition, we found upregulation of characteristic markers from the three branches of the ER stress response system that are activated by GRP78, including the protein kinase RNA-activated-like ER kinase (PERK) branch, the activating transcription factor 6 (ATF6) branch, and the Inositol-Requiring Enzyme 1 (IRE1) branch. Furthermore, transcriptional expression of apoptosis markers such as C-EBP-Homologous Protein (CHOP) and caspase 9 was highly increased, indicating that ER stress in *scfd1*^vcc44−/−^ embryos was long-standing and apoptosis likely initiated (Figure 5E). Taken together, these results show that there were significant, sustained perturbations of ER function in *scfd1*^vcc44−/−^ embryos, which likely contribute to the disruption of normal cardiac development and function in these fish.

### 3.6. Partial Scfd1 Deficiency Leads to Mild Cardiac Dysfunction in Adult Zebrafish

**Figure 6:**
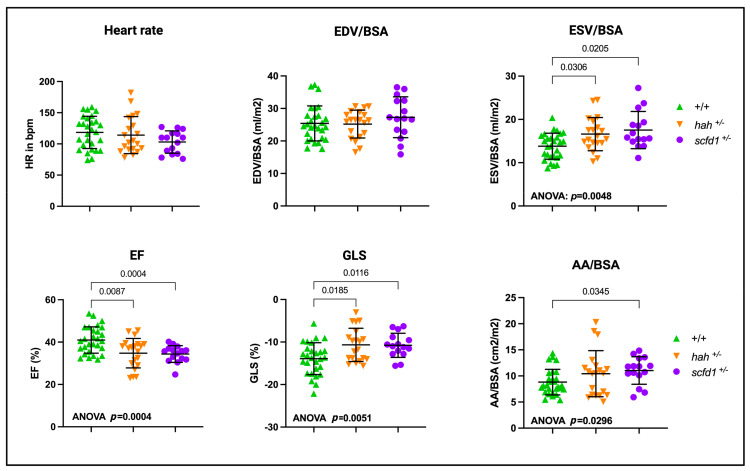
*scfd1* deficiency leads to systolic dysfunction in adult zebrafish hearts. Scatter graphs showing high-frequency echocardiography data from heterozygous *hah^vcc43+/−^* and *scfd1^vcc44+/−^* mutants aged 9–15 months. Heart rate and ventricle end-diastolic volume (EDV), normalized to body surface area, (BSA) were normal, while ventricle end-systolic volume (ESV), normalized to BSA, was increased. Ventricular ejection fraction (EF) and global longitudinal strain (GLS) were reduced in *hah^vcc43+/−^* and *scfd1^vcc44+/−^* mutants in comparison to wildtype (+/+). Maximal atrial area (AA) was increased in *scfd1^vcc44+/−^* mutants. Significant one-way ANOVA and multiple comparisons *p*-values stated. Significance if *p* < 0.05. Created with BioRender.com.

Given that many genetic cardiomyopathies manifest in heterozygous variant carriers as adult-onset disease, we explored the possibility that heterozygous *scfd1* deficiency would lead to cardiac dysfunction in adult fish. To do this, we performed high-frequency echocardiography in both *hah*^vcc43+/−^ and *scfd1*^vcc44+/−^ adults at 9–15 months of age (Figure 6). Interestingly, while BSA-indexed ventricular EDV was still preserved, indexed ESV was increased in both *hah*^vcc43+/−^ and *scfd1*^vcc44+/−^ mutants (two-way ANOVA, *p* = 0.005, with multiple comparisons: WT, 13.82 mL/m^2^ vs. *hah*^vcc43+/−^, 16.60 mL/m^2^, *p* = 0.031; WT vs. *scfd1*^vcc44+/−^, 17.54 mL/m^2^, *p* = 0.021). Accordingly, both mutants showed significant contractile dysfunction, as indicated by reduced EF (two-way ANOVA (*p* = 0.0004) with multiple comparisons: WT, 40.99% vs. *hah*^vcc43+/−^, 34.78%, *p* = 0.009; WT vs. *scfd1*^vcc44+/−^ 34.39%, *p* = 0.0004) and reduced (less negative) GLS (two-way ANOVA (*p* = 0.005) with multiple comparisons: WT, −13.92% vs. *hah*^vcc43+/−^, −10.67%, *p* = 0.019; WT vs. *scfd1*^vcc44+/−^ −10.77%, *p* = 0.012). Atrial size was mildly increased in *scfd1*^vcc44+/−^ mutants (AA, two-way ANOVA (*p* = 0.030) with multiple comparisons: WT, 8.82 cm^2^/m^2^ vs. *scfd1*^vcc44+/−^, 11.04 cm^2^/m^2^, *p* = 0.002). Heart rate was not different between the groups. Taken together, these findings indicate that heterozygous Scfd1 deficiency causes DCM-like ventricular systolic dysfunction in adult fish. Atrial dilation could be related to ventricular defects or could potentially be indicative of a primary atrial myopathic process. There was a trend towards a slightly more pronounced phenotype in *scfd1*^vcc44+/−^ mutants, which likely reflects disease progression with age, as these fish were 6 months older than *hah*^vcc43+/−^ mutants at the time of examination. There were no gross craniofacial abnormalities or pericardial edema in both adult *scfd1*^vcc44+/−^ and *hah*^vcc43+/−^ fish.

## 4. Discussion

Here, we describe the mapping of a novel zebrafish heart failure mutant, *hah*^vcc43^, with multiple lines of evidence indicating that a loss-of-function variant in the highly conserved sec1 family domain-containing 1 gene, *scfd1*, is underlying this phenotype. These include: (i) Scfd1 is expressed in the zebrafish heart, (ii) Scfd1 expression is lost in *hah*^vcc43^ mutants, (iii) the *hah*^vcc43^ phenotype is copied by MO-mediated knockdown or CRISPR/Cas9-mediated knock-out of *scfd1*, and (iv) the *hah*^vcc43^ phenotype is partially rescued by WT *scfd1* mRNA injection. Using functional and ultrastructural analysis, we show that complete *scfd1* deficiency in embryos causes loss of cardiac contractility, reduced cardiomyocyte sarcomere content, and ER stress; findings that are, in part, replicated in adults with heterozygote *scfd1* deficiency. Our results provide new insights into the key role of *scfd1* in cardiac structure and function and highlight the importance of ER stress in the pathogenesis of heart disease.

Two zebrafish *scfd1* mutants have previously been described. Hou et al. [19] recovered a zebrafish *scfd1* null mutant from a gene trapping screen, which showed craniofacial abnormalities similar to *hah*^vcc43^. The authors discovered an essential role of Scfd1 in cartilage formation via the protein’s function in chondrocyte ER-Golgi-mediated secretion of procollagen II, a role that was conserved in mammals. Of note, heart function was not evaluated in that study. Nechiporuk et al. [20] independently isolated the zebrafish *scfd1* mutant *emmental* (*emm*) from a mutagenesis screen designed to produce temperature-sensitive mutants that have stage-specific defects in caudal fin regeneration. *emm* was found to carry a splice site mutation after exon 5 of *scfd1* that resulted in the generation of two mutant transcripts from two cryptic splice sites. The resulting mutant gene products were predicted to alter the important conserved sec1 domain of Scfd1, likely leading to at least partial loss of function. *emm* mutants displayed a regenerative block during the formation of the blastema, a mass of pluripotent mesenchymal cells required for the later stages of fin regeneration. Blastemal cells exhibited Scfd1-mediated vesicular transport defects and distended ER lumina consistent with ER stress, which ultimately led to mesenchymal cell apoptosis. *emm* embryos were described as having dysmorphic hearts, but cardiac structure and function were not specifically characterized.

Here, we describe two new *scfd1* alleles, *hah*^vcc43^ and *scfd1*^vcc44^, and specifically focus on the heart, a tissue that is—in contrast to chondrocytes and blastemal cells—not highly secretory or proliferative. Both new *scfd1* alleles carry PTCs in exon 10 and are likely loss-of-function mutants. Orthologous human SCFD1 PTCs produce a NMDetective-A [39] score of 0.63, suggesting that they efficiently trigger nonsense-mediated decay (NMD) of mutant transcripts. That NMD of mutant *scfd1* transcripts also occurs in zebrafish is supported by our Western blot results, which show that Scfd1 is completely lost in homozygous animals and that there is no significant expression of truncated protein species. That *scfd1* transcript levels appear only partially reduced using qPCR could reflect compensatory upregulation of *scfd1* transcription in response to Scfd1 protein deficiency. Further evidence in support of *hah*^vcc43^ and *scfd1*^vcc44^ being loss-of-function mutants is provided by *scfd1* morphants copying *hah*^vcc43^ and *scfd1*^vcc44^ mutant phenotypes, including the cartilage phenotype present in the published *scfd1* null mutant [19].

Coupling of SM proteins to SNARE complexes is almost universally required for membrane fusion. Scfd1 was the first SM protein cloned nearly three decades ago in the yeast *Saccharomyces cerevisiae*, where it was found to have an essential function in vesicular transport and secretion [15,40,41]. Subsequent studies in yeast and mammalian cells showed that Scfd1 binds to SNAREs such as syntaxins STX5, STX17, and STX18 to regulate ante- and retrograde ER-Golgi transport via vesicles coated in cage protein complexes COPI and COPII [42,43]. Recent studies in zebrafish and mammalian cells have furthermore indicated an important role of Scfd1 in the formation of large, COP-independent ER transport vesicles involved in the export of bulky extracellular matrix (ECM) collagens, via interaction with STX18 and TANGO1 [19,44].

Our data show that Scfd1 is indispensable for normal heart development and function in zebrafish. In *scfd1* mutants, the first steps of heart development, such as the formation of the heart tube, initiation of peristaltic contractions, and cardiac chamber specification and looping, seemed to progress relatively normally between 1–2 dpf. This may, in part, be due to the presence of maternal gene products. However, from 3 dpf onwards, ventricular function rapidly declined, and chamber maturation and ballooning did not progress, as evidenced by the reduced ventricular wall thickness and end-diastolic size, respectively. In addition, there was progressive loss of contractility and embryonic lethality. To investigate the cause of cardiac dysfunction in our mutants, we undertook detailed electron microscopic analysis of WT and *scfd1*^vcc44−/−^ hearts. Indeed, we found thin ventricular walls, as well as altered myofibril ultrastructure and reduced sarcomere content in Scfd1-deficient cardiomyocytes. These defects likely contributed to the contractile impairment of *scfd1*^vcc44^ hearts. Because vigorous cardiac contractions and blood flow are required for normal chamber maturation [45], the lack of these hemodynamic stimuli in *scfd1*^vcc44−/−^ fish may have enhanced the observed morphological heart defects further.

Given Scfd1’s proposed role in vesicular transport in other cell types, we next examined the reticular and Golgi networks in WT and *scfd1*^vcc44−/−^ cardiomyocytes. Strikingly, we found ER/SR disorganization, with altered localization and size variation of tubules and increased membrane-bound and free ribosome density outside the perinuclear area, as well as highly vesiculated Golgi stacks. This is indicative of a severe disruption of the cell’s endo-membrane and protein handling systems. Similar ultrastructural defects were also observed in non-cardiomyocyte interstitial cells in the heart and resemble the ER defects found in *scfd1*-deficient zebrafish chondrocytes and blastema cells [19,20], as well as yeast [40]. Fittingly, altered expression of the Scfd1 binding partners, TANGO1 and STX5, has previously been reported to cause Golgi fragmentation [46] and ER distention [47] in mammalian cell models and zebrafish, respectively. Whether expression of these binding partners is altered in our mutants remains to be investigated. While our findings suggest that the cardiomyocyte changes in *scfd1*^vcc44−/−^ mutants occur in a cell-autonomous manner, we cannot exclude that non-autonomous mechanisms contribute to the observed heart phenotype.

We hypothesized that the structural changes in the endomembrane system would lead to perturbations of ER function and an activation of the UPR system. This highly conserved ER signaling system is characterized by upregulation of the synthesis of chaperone proteins, a decrease in general protein synthesis, and ERAD activation, which eliminates misfolded proteins by translocation to the cytoplasm where they are ubiquitinated to target them for proteasomal degradation [7,8,37,38]. On the molecular level, three key stress signaling pathways are activated when ER chaperones, such as GRP78, dissociate from ER transmembrane anchors. Activation of PERK leads to phosphorylation of eIF2, which blocks mRNA translation and reduces protein synthesis. Activation of ATF6 promotes expression of XBA-1 and CHOP, and, similarly, activation of IRE-1 also activates XBP-1, both leading to transcriptional activation of ER chaperones and ERAD proteins to enhance removal of misfolded/accumulated proteins [7,8,37,38]. While activation of the UPR is aimed at restoring homeostasis, persistent ER stress eventually activates the caspase cascade and triggers apoptosis [48,49,50]. Indeed, in *scfd1*-deficient mutants, we found transcriptional upregulation of the zebrafish orthologues of all key UPR genes, as well as of apoptosis markers, indicating that persisting ER stress plays a major part in the cardiac phenotype these fish display. Our findings are in line with studies in zebrafish and mouse models that have described cardiac developmental and functional defects due to ER stress caused by the loss of specific ER resident proteins [51,52]. More generally, ER stress has been proposed to be involved in the pathogenesis of many human cardiovascular disorders, including myocardial infarction, cardiac hypertrophy, DCM, and heart failure [6,7,8]. Likewise, abnormal protein handling and degradation plays an important role in cardiomyopathy development due to mutations in genes such as *BAG3* and *FLNC* [4,5]. We note that our transcriptional analyses were performed using whole embryo samples due to the technical difficulties involved in embryonic heart isolation. Therefore, the upregulation of ER stress markers is likely due to activation of stress response pathways, not only in the heart, but also in cartilage and other tissues that express *scfd1*. However, given the striking ultrastructural Golgi/ER defects evident in cardiomyocytes, it appears likely that ER stress in the heart contributes to our qRT-PCR findings.

There are several possibilities for how ER/Golgi defects and subsequent ER stress might lead to systolic dysfunction in the heart on a mechanistic level. For example, vesicular transport defects could interrupt transport of contractile proteins to the sarcomeres, cardiac ion channel proteins to the sarcolemma, or the secretion of ECM proteins and cardiokines, leading to primary or secondary effects on cardiac contractility. Another possibility involves the observed activation of the UPR in *scfd1* mutants, which is predicted to lead to a general downregulation of protein translation in favor of upregulation of ER stress response factors. In cardiomyocytes, this might reduce translation of sarcomeric proteins, which could contribute to the decreased sarcomere density and myofibril width observed in *scfd1* embryonic mutant hearts. In addition, translation of proteins involved in mitochondrial biogenesis and respiration could be reduced, leading to mitochondrial dysfunction and altered energetics, which could contribute to contractile dysfunction. Further studies will be required to characterize the specific proteins dysregulated by *scfd1*-deficiency in the heart. Prola et al. demonstrated that ER stress in mouse cardiomyocytes induced a reorganization of the reticular endomembrane system, very similar to what we observed in *scfd1* mutants, and wide-reaching alterations of mitochondrial function [36]. Whether mitochondrial dysfunction contributes to the cardiac phenotype observed in *scfd1* mutants will require further investigation. In the context of the predicted general arrest of protein translation during ER stress, it might seem counterintuitive that we observed an increase rather than a decrease in membrane-bound and free ribosomes in the intermyofibrillar region. However, a continued association of ER-bound ribosomes as a consequence of UPR induction has been reported [53], and Prola et al. found an increased presence of rough instead of smooth reticulum at the interfibrillar space and in the vicinity of mitochondria in mouse cardiomyocytes [36], similar to what we observed. Another factor that might contribute to contractile dysfunction in our mutants is cardiomyocyte loss due to the activation of apoptosis after long-standing ER stress. While cardiomyocyte apoptosis remains to be further investigated using immunohistochemistry, this could explain why ventricles appear thin-walled. Lastly, the presence of reticular network disorganization also in interstitial cells of the heart, not just cardiomyocytes, raises the possibility that, for example, fibroblast dysfunction and altered ECM content and composition due to collagen export defects, as reported for *scfd1*-deficient chondrocytes [19], might further contribute to the heart phenotype of *scfd1* mutants. While fibrosis and ECM changes are known secondary characteristics of many cardiovascular disorders, in particular cardiomyopathies, a potential primary role in disease development remains to be explored.

In contrast to the severe, embryonic lethal heart phenotype in homozygous *scfd1*^vcc44−/−^ mutants, heterozygous *scfd1*^vcc44+/−^ fish showed a mild adult-onset DCM phenotype. Mechanistically, reduced Scfd1 levels in heterozygous mutants might lead to mild endo-membrane dysfunction and low-level activation of ER stress responses. Alternatively, long-standing *scfd1* deficiency during embryonic and larval stages might have formed a developmental template for poor proteostasis and metabolic function, ultimately leading to cardiac dysfunction with age. A comprehensive evaluation of heart structure and function in adult *scfd1* fish, for example, under stress conditions, is warranted but beyond the scope of this study. However, the finding that even partial *scfd1* loss can cause significant systolic dysfunction in adulthood highlights the importance of *scfd1* for normal heart function in zebrafish, a role that might be conserved in humans. Zebrafish *scfd1* and human SCFD1 share 88% amino acid identity [20]. This sequence conservation, and the fact that the role of *scfd1* in zebrafish chondrogenesis and cartilage collagen exportation is conserved in mammals [19], suggest that its role in cardiomyocyte function might also be conserved. Although further studies are required to specifically evaluate the role of *scfd1* in mature heart function, our data suggest that *scfd1* deficiency and altered Golgi/ER function could have a primary role in the pathogenesis of human DCM.

## 5. Conclusions

In conclusion, our work provides new insights into the indispensable role of *scdf1* for normal cardiac development and function. Our data point to the importance of the ER/Golgi system in cardiomyocyte biology and identify components of this pathway as candidate genes for inherited cardiomyopathies and novel targets for therapy.

## Data Availability

Study data and materials are available from the corresponding author upon reasonable request.

## Supplementary Materials

The following supporting information can be downloaded at: https://www.mdpi.com/article/10.3390/jcdd10100408/s1, Figure S1: Very high evolutionary conservation of Scfd1; Figure S2: Reduced Scfd1 protein expression in *hah^vcc43^* and *scfd1^vcc44^* mutants at 3 dpf; Figure S3: Gill chondrocyte defects in *scfd1^vcc44−/−^* mutants at 3 dpf; Figure S4: Homozygous *scfd1^vcc44−/−^* mutants have normal skeletal muscle ultrastructure at 3dpf; Table S1: Primer sets used in qPCR analysis; Table S2: Assessment of cardiac size and function in embryonic wildtype and *scfd1* mutant fish. References [20,54,55] are cited in the Supplementary Materials.
