# Supplementary material for: Loss of Sec-1 Family Domain-Containing 1 (scfd1) Causes Severe Cardiac Defects and Endoplasmic Reticulum Stress in Zebrafish
Source: J Cardiovasc Dev Dis. 2023 Sep 22;10(10):408. doi: 10.3390/jcdd10100408 (PMC10607167; doi:10.3390/jcdd10100408)
Supplement: Supplementary file 1 [file jcdd-10-00408-s001.zip › jcdd-2339484-supplementary.pdf]

Loss of Sec-1 Family Domain-containing 1 (*scfd1*) Causes Severe Cardiac Defects and Endoplasmic Reticulum Stress in Zebrafish

SUPPLEMENTAL MATERIAL

Item	Title	Page no.
Supplemental Figure S1	Very high evolutionary conservation of Scfd1.	2
Supplemental Figure S2	Reduced Scfd1 protein expression in *hah*^{vcc43} and *scfd1*^{vcc44} mutants at 3 dpf.	3
Supplemental Figure S3	Gill chondrocyte defects in *scfd1*$^{vcc44-/-}$ mutants at 3 dpf.	4
Supplemental Figure S4	Homozygous *scfd1*$^{vcc44-/-}$ mutants have normal skeletal muscle ultrastructure at 3dpf.	5
Supplemental Table S1	Primer sets used in qPCR analysis.	6
Supplemental Table S2	Assessment of cardiac size and function in embryonic wildtype and *scfd1* mutant fish.	7
Supplemental References		8

```
Q8WVM8 SCFD1_HUMAN            1 MAAAAAATAAAAASIRERQTVALKRMLNFNVPHIKNSTGEPVWKVLIYDRFGQDIISPLL       60
Q8BRF7 SCFD1_MOUSE            1 ---MVGSKMAATASIRERQTVALKRMLNFNVPHVKNSTGEPVWKVLIYDRFGQDIISPLL       57
B3DKI8 B3DKI8_DANRE           1 ----------MAASIREKQTAALKRMLNFNAPPLKNTTAEPVWKVLIYDRFGQDIISPLL       50
                                           :*****:**.*********.* :**:*.*********************

Q8WVM8 SCFD1_HUMAN           61 SVKELRDMGITLHLLLHSDRDPIPDVPAVYFVMPTEENIDRMCQDLRNQLYESYYLNFIS      120
Q8BRF7 SCFD1_MOUSE           58 SVKELRDMGITLHLLLHSDRDPIPDVPAVYFVMPTEENIDRLCQDLRNQLYESYYLNFIS      117
B3DKI8 B3DKI8_DANRE          51 SVKELRDMGITLHLLLHSDRDPIPDVPAIYFIMPTEENIDRICQDLRNQLYESYYLNFIS      110
                                ****************************:**:*********:******************

Q8WVM8 SCFD1_HUMAN          121 AISRSKLEDIANAALAASAVTQVAKVFDQYLNFITLEDDMFVLCNQNKELVSYRAINRPD      180
Q8BRF7 SCFD1_MOUSE          118 AISRSKLEDIANAALAASAVTQVAKVFDQYLNFITLEDDMFVLCNQNKELVSYRAINRPD      177
B3DKI8 B3DKI8_DANRE         111 AISRSKLEDIASAALAANAVNQVTKVFDQYLNFITLEDDMFILCNQNKEHISYHAINKPD      170
                                ***********.*****.**.**:*****************:******* :**:***:**

Q8WVM8 SCFD1_HUMAN          181 ITDTEMETVMDTIVDSLFCFFVTLGAVPIIRCSRGTAAEMVAVKLDKKLRENLRDARNSL      240
Q8BRF7 SCFD1_MOUSE          178 ITDTEMETVMDTIVDSLFCFFVTLGAVPIIRCSRGTAAEMVAVKLDKKLRENLRDARNSL      237
B3DKI8 B3DKI8_DANRE         171 IMDTDMEGIMDTIVDSLFCFFVTLGAVPIIRCPRGNAAEMVAVKLDKKLRENLRDARNSL      230
                                * **:** :*********************** **.************************

Q8WVM8 SCFD1_HUMAN          241 FTGDTLGAGQFSFQRPLLVLVDRNIDLATPLHHTWTYQALVHDVLDFHLNRVNLEESSGV      300
Q8BRF7 SCFD1_MOUSE          238 FTGDPLGTGQFSFQRPLLVLVDRNIDLATPLHHTWTYQALVHDVLDFHLNRVNLEESTGV      297
B3DKI8 B3DKI8_DANRE         231 FTGDTMGTGQFSFQRPLFVLADRNLDLATPLHHTWTYQALIHDVLDFHLNRVSVDESHGS      290
                                **** :*:*********:**.***:***************:***********.::** * 

Q8WVM8 SCFD1_HUMAN          301 ENSPAGARPKRKNKKSYDLTPVDKFWQKHKGSPFPEVAESVQQELESYRAQEDEVKRLKS      360
Q8BRF7 SCFD1_MOUSE          298 ENSPAGARPKRKNKKSYDLTPVDKFWQKHKGSPFPEVAESVQQELESYRAQEDEVKRLKS      357
B3DKI8 B3DKI8_DANRE         291 EASPAGARPKKKNKKSYDLTAADKFWQKHKGSPFPEVAEAVQEELDTYRAQEDEVKRLKS      350
                                * ********:********* .*****************:**:**::*************

Q8WVM8 SCFD1_HUMAN          361 IMGLEGEDEGAISMLSDNTAKLTSAVSSLPELLEKKRLIDLHTNVATAVLEHIKARKLDV      420
Q8BRF7 SCFD1_MOUSE          358 IMGLEGEDEGAISMLSDNTAKLTSAVSSLPELLEKKRLIDLHTNVATAVLEHIKARKLDV      417
B3DKI8 B3DKI8_DANRE         351 IMGLEGEDDGAISMLSDNTAKLTSAVSSLPELLEKKRLIDLHTNVATAVLDHIKSRKLDV      410
                                ********:*****************************************:***:*****

Q8WVM8 SCFD1_HUMAN          421 YFEYEEKIMSKTTLDKSLLDIISDPDAGTPEDKMRLFLIYYISTQQAPSEADLEQYKKAL      480
Q8BRF7 SCFD1_MOUSE          418 YFEYEEKIMSKTTLDKSLLDVISDPDAGTPEDKMRLFLIYYISAQQAPSEVDLEQYKKAL      477
B3DKI8 B3DKI8_DANRE         411 YFEYEEKLMSKSTLDKSLLDIISDPDAGTAEDKMRLFLIYYITSQQPPLEADLEQYKKAM      470
                                *******:***:********:******** ************::** * *.********:

Q8WVM8 SCFD1_HUMAN          481 TDAGCNLNPLQYIKQWKAFTKMASAPASYGSTTTKPMGLLSRVMNTGSQFVMEGVKNLVL      540
Q8BRF7 SCFD1_MOUSE          478 TDAGCNLSPLQYIKQWKAFAKMASTPASYGNTTTKPMGLLSRVMNTGSQFVMEGVKNLVL      537
B3DKI8 B3DKI8_DANRE         471 VDAGCDLSPLNYIKQWKAFTKMAATPANYGSSGVKPMGLFSRVMNTGSQFVMEGVKNLVL      530
                                .****:*.**:********:***::**.**.: .*****:********************

Q8WVM8 SCFD1_HUMAN          541 KQQNLPVTRILDNLMEMKSNPETDDYRYFDPKMLRGNDSSVPRNKNPFQEAIVFVVGGGN      600
Q8BRF7 SCFD1_MOUSE          538 KQQNLPVTRILDNLMEMKSNPETDDYRYFDPKMLRSNDSSVPRNKSPFQEAIVFVVGGGN      597
B3DKI8 B3DKI8_DANRE         531 KQHNLPVTRILDNLMEMKSNPETDDYRYFDPKMLRGSESSIPRNKNPFQEAIVFVVGGGN      590
                                **:********************************..:**:****.**************

Q8WVM8 SCFD1_HUMAN          601 YIEYQNLVDYIKGKQGKHILYGCSELFNATQFIKQLSQLGQK                        642
Q8BRF7 SCFD1_MOUSE          598 YIEYQNLVDYIKAKQGKHILYGCSEIFNATQFIKQLSQLGQK                        639
B3DKI8 B3DKI8_DANRE         591 YIEYQNLVDYTKTRQGKRILYGCSELFNAAQFIKQLSQLGQK                        632
                                ********** * :***:*******:***:************
```

Supplemental Figure S1. Very high evolutionary conservation of Scfd1. Alignment of the protein sequences of human (Q8WVM8) SCFD1, mouse (Q8BRF7) SCDF1 and zebrafish (B3DKI8) Scfd1, generated using the UniProt website (uniprot.org). Epitope against which the SCFD1 antibody was raised is underlined in blue. Amino acid affected by *hah*^{vcc43} PTC indicated by red box. Zebrafish and human SCFD1 share 88% amino acid identity (1). Created with BioRender.com.

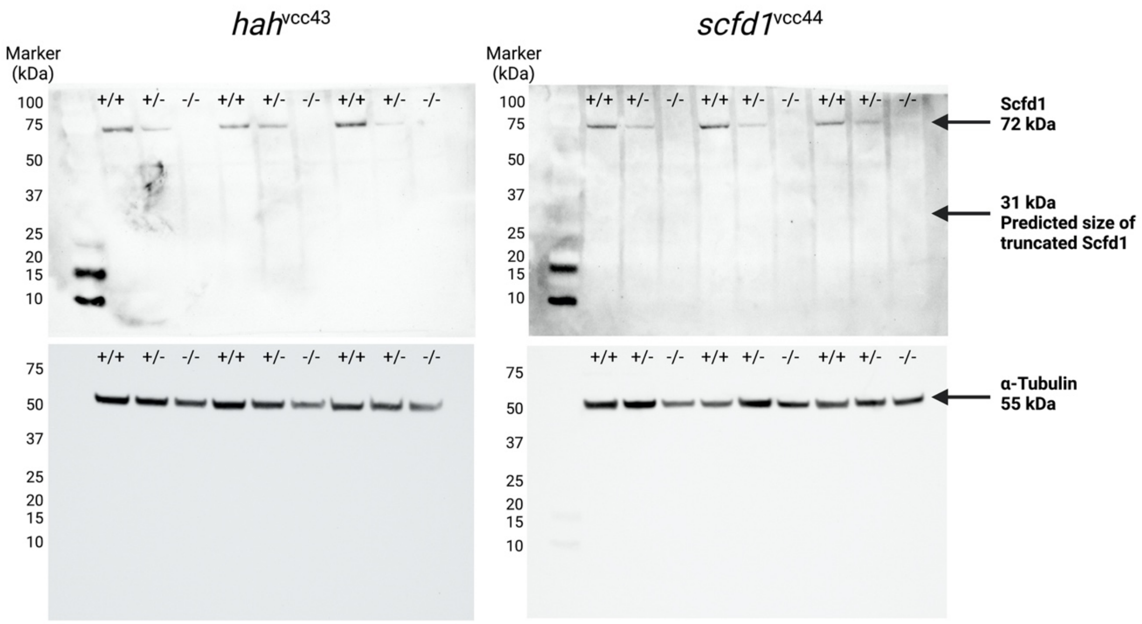

Supplemental Figure S2. Reduced Scfd1 protein expression in *hah^{vcc43}* and *scfd1^{vcc44}* mutants at 3dpf. Western Blots of wildtype (+/+), heterozygous (+/-) and homozygous (-/-) *hah^{vcc43}* (left) and *scfd1^{vcc44}* (right) mutant samples at 3dpf (30 pooled embryos/sample), stained with either Scfd1(top) or α-Tubulin (bottom) antibodies. Molecular weights of full length and truncated Scfd1, as well as α-Tubulin, indicated (black arrows) relative to protein marker. Created with BioRender.com.

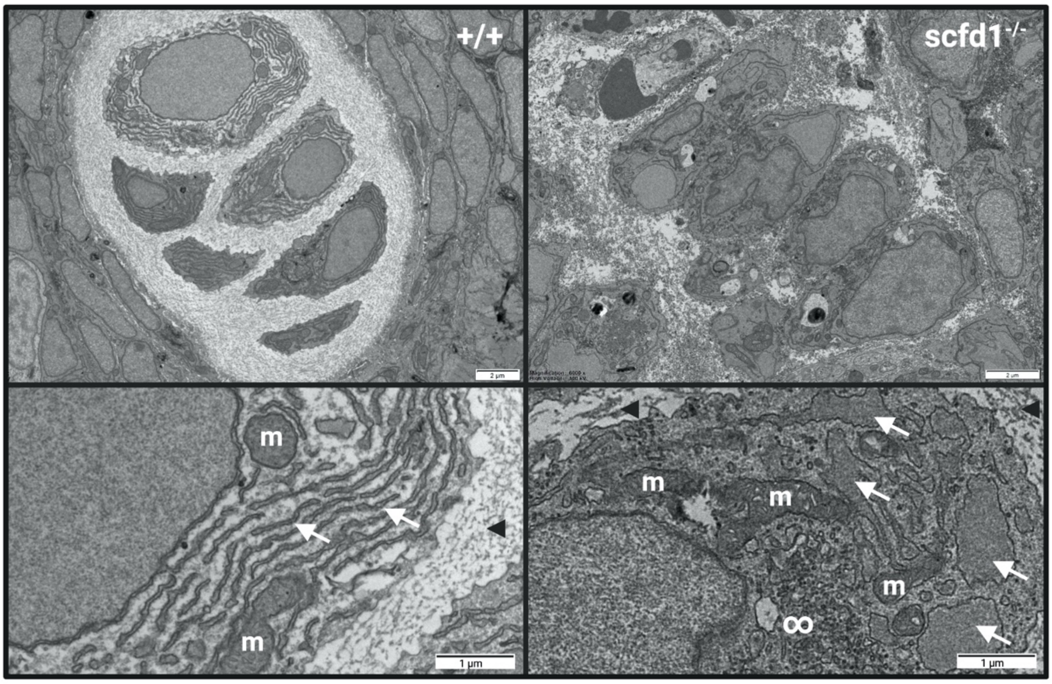

Supplemental Figure S3. Gill chondrocyte defects in *scfd1*$^{vcc44\ -/-}$ mutants at 3 dpf. Low and high magnification electron micrographs showing gill chondrocyte defects in *scfd1$^{vcc44-/-}$* mutant embryos (right) in comparison to wildtype (+/+; left). White arrows indicate well-stacked ER in +/+ vs. distended ER membranes as well as accumulation of vesicles (∞) in *scfd1*$^{vcc44\ -/-}$ embryos. Black arrow heads point to regular extracellular matrix (ECM) collagen fibrils in +/+ vs. reduced ECM with granular fibril appearance in *scfd1*$^{vcc44-/-}$ embryos; m=mitochondrium. Created with BioRender.com.

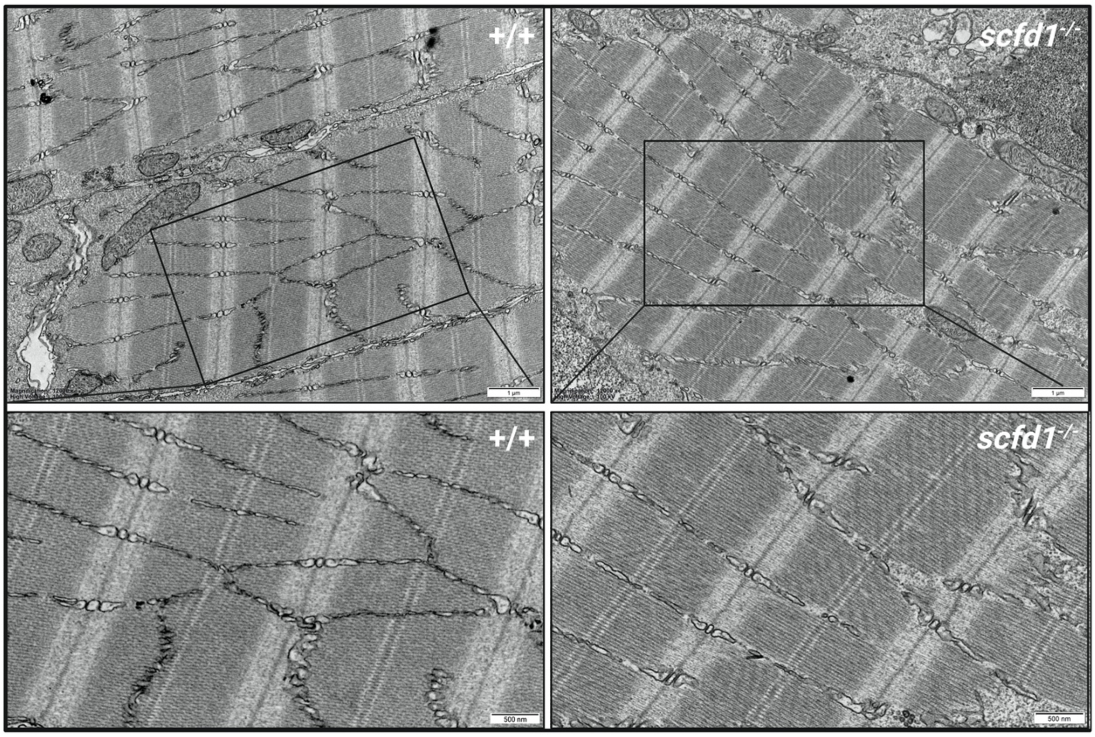

Supplemental Figure S4. Homozygous *scfd1$^{vcc44-/-}$* mutants have normal skeletal muscle ultrastructure at 3 dpf. Representative low (top panels) and high magnification (bottom panels) electron micrographs of skeletal muscle in WT (+/+) and *scfd1$^{vcc44-/-}$* mutant embryos.

Supplemental Table S1. Primer sets used in qPCR analysis.

Gene	Forward (5'-3')	Reverse (5'-3')	Reference
tmem50a	ATTTGTGTTGATTTTGCTGTCG	TTCTGTTGCAAACTCTGAGGAA	this publication
ube2a	TGACTGTTGACCCACCTTACAG	CAAATAAAAGCAAGTAACCCCG	this publication
scfd1	TCGATGTCCAAGAGGGAAT	ACTTGTCAGCAGCAGTGAGA	this publication
hspa5	AAGAGGCCGAAGAGAAGGAC	AGCAGCAGAGCCTCGAAATA	[2]
eif2ak3	TGGGCTCTGAAGAGTTCGAT	TGTGAGCCTTCTCCGTCTTT	[2]
atf4a	CTTTCTCTCCTCCTGCTTCT	GAGTCACACGACCCAATCA	[2]
ern1	TGACGTGGTGGAAGTTGGTA	ACGGATCACATTGGGATGTT	[2]
atf6	CTGTGGTGAAACCTCCACCT	CATGGTGACCACAGGAGATG	[2]
eif2s1	CCAAAGATGAGCAGCTGGAGA	ATCCGACACAGCCTGCTTAAA	[3]
xbp-1s	TGTTGCGAGACAAGACGA	CCTGCACCTGCTGCGGACT	[3]
chop	GAGTTGGAGGCGTGGTATGA	CCTTGGTGGCGATTGGTGAA	[3]
caspase 9	AAATACATAGCAAGGCAACC	CACAGGGAATCAAGAAAGG	[3]

Supplemental Table S2. Assessment of cardiac size and function in embryonic wildtype and *scfd1* mutant fish.

**Variable**	+/+		***hah***$^{+/-}$			***hah***$^{-/-}$			***scfd1***$^{+/-}$			***scfd1***$^{-/-}$		
**Heart rate (bpm)**	**Mean ± SD**	**N**	**Mean ± SD**	**N**	***P*** ***(vs. +/+)***	**Mean ± SD**	**N**	***P*** ***(vs. +/+)***	**Mean ± SD**	**N**	***P*** ***(vs. +/+)***	**Mean ± SD**	**N**	***P*** ***(vs. +/+)***
3 dpf	153.4 ± 10.0	26	158.1 ± 7.8	30	0.996	132.2 ± 15.1	35	0.429	148.2 ± 7.6	23	0.996	139.7 ± 9.4	21	0.880
4 dpf	152.8 ± 9.2	26	156.5 ± 8.7	30	0.998	127.8 ± 10.4	35	0.375	148.0 ± 10.8	22	0.997	129.0 ± 14.4	18	0.601
5 dpf	159.8 ± 8.9	26	157.1 ± 10.8	30	0.999	107.4 ± 20.5	24	0.009	151.8 ± 14.7	21	0.984	85.7 ± 25.1	16	0.004
**Ventricle FAC (%)**					***P*** ***(vs. +/+)***			***P*** ***(vs. +/+)***			***P*** ***(vs. +/+)***			***P*** ***(vs. +/+)***
3 dpf	33.3 ± 4.0	15	27.5 ± 3.0	18	0.057	22.2 ± 6.6	20	<0.0001	32.5 ± 4.3	23	0.872	28.5 ± 4.9	16	0.013
4 dpf	26.5 ± 8.2	15	24.3 ± 3.7	18	0.251	14.7 ± 4.7	16	<0.0001	23.4 ± 5.4	22	0.099	13.1 ± 9.7	16	<0.0001
5 dpf	21.4 ± 8.1	15	21.5 ± 2.6	14	0.981	4.7 ± 4.1	14	<0.0001	24.2 ± 4.5	21	0.596	2.1 ± 3.1	12	<0.0001
**Ventricle EDA (mm^2)**					***P*** ***(vs. +/+)***			***P*** ***(vs. +/+)***			***P*** ***(vs. +/+)***			***P*** ***(vs. +/+)***
3 dpf	0.035 ± 0.004	15	0.033 ± 0.006	18	0.716	0.031 ± 0.006	20	0.101	0.036 ± 0.003	23	0.732	0.035 ± 0.003	16	0.991
4 dpf	0.031 ± 0.006	15	0.030 ± 0.006	18	0.606	0.027 ± 0.004	16	0.085	0.028 ± 0.004	22	0.475	0.025 ± 0.004	16	0.0012
5 dpf	0.031 ± 0.006	15	0.029 ± 0.005	14	0.317	0.020 ± 0.004	14	<0.0001	0.030 ± 0.005	21	0.691	0.022 ± 0.004	12	<0.0001

EDA, ventricular end-diastolic volume; ESA, ventricular end-systolic volume; FAC, ventricular fractional area change {(EDA-ESA)/EDA}. Data are reported as mean ± SD.For each variable, 2-way ANOVA with multiple comparisons was performed, with time factor, genotype factor and interaction between both factors all significant (p<0.0001, respectively). Individual comparisons to wildtype (+/+) reference values listed; significant p-values highlighted in blue. Significance if $p < 0.05$.

Supplemental References

1. Nechiporuk A, Poss KD, Johnson SL, Keating MT. *Positional cloning of a temperature-sensitive mutant emmental reveals a role for sly1 during cell proliferation in zebrafish fin regeneration.* Dev Biol. 2003 Jun 15;258(2):291-306. doi: 10.1016/s0012-1606(03)00129-5.
2. Zhang M, Chen J, Jiang Y, Chen T. *Fine particulate matter induces heart defects via AHR/ROS-mediated endoplasmic reticulum stress.* Chemosphere. 2022 Nov;307(Pt 2):135962. doi: 10.1016/j.chemosphere.2022.135962.
3. Qi M, Dang Y, Xu Q, Yu L, Liu C, Yuan Y, Wang J. *Microcystin-LR induced developmental toxicity and apoptosis in zebrafish (Danio rerio) larvae by activation of ER stress response.* Chemosphere. 2016 Aug;157:166-73. doi: 10.1016/j.chemosphere.2016.05.038.
